# Novel Cysteine Protease Inhibitor Derived from the *Haementeria vizottoi* Leech: Recombinant Expression, Purification, and Characterization

**DOI:** 10.3390/toxins13120857

**Published:** 2021-12-02

**Authors:** Débora do Carmo Linhares, Fernanda Faria, Roberto Tadashi Kodama, Adriane Michele Xavier Prado Amorim, Fernanda Calheta Vieira Portaro, Dilza Trevisan-Silva, Karla Fernanda Ferraz, Ana Marisa Chudzinski-Tavassi

**Affiliations:** 1 Laboratory of Industrial Biotechnology, Institute for Technological Research, Av. Prof. Almeida Prado, 532, São Paulo 05508-901, SP, Brazil; deboradclinhares@gmail.com; 2 Laboratory of Development and Innovation, Butantan Institute, Av. Vital Brasil, 1500-Butantã, São Paulo 05503-900, SP, Brazil; adriane_mi@yahoo.com.br (A.M.X.P.A.); karla.ferraz@esib.butantan.gov.br (K.F.F.); 3 Laboratory of Biomolecule Structure and Function, Butantan Institute, Av. Vital Brasil, 1500-Butantã, São Paulo 05503-900, SP, Brazil; pararoberval@gmail.com (R.T.K.); fernanda.portaro@butantan.gov.br (F.C.V.P.); 4 Centre of Excellence in New Target Discovery—CENTD, Butantan Institute, Av. Vital Brasil, 1500-Butantã, São Paulo 05503-900, SP, Brazil; dilza.silva@butantan.gov.br

**Keywords:** leech, *Haementeria vizottoi*, cysteine proteases inhibitor, recombinant cystatin, cathepsin L

## Abstract

Cathepsin L (CatL) is a lysosomal cysteine protease primarily involved in the terminal degradation of intracellular and endocytosed proteins. More specifically, in humans, CatL has been implicated in cancer progression and metastasis, as well as coronary artery diseases and others. Given this, the search for potent CatL inhibitors is of great importance. In the search for new molecules to perform proteolytic activity regulation, salivary secretions from hematophagous animals have been an important source, as they present protease inhibitors that evolved to disable host proteases. Based on the transcriptome of the *Haementeria vizzotoi* leech, the cDNA of Cystatin-Hv was selected for this study. Cystatin-Hv was expressed in *Pichia pastoris* and purified by two chromatographic steps. The kinetic results using human CatL indicated that Cystatin-Hv, in its recombinant form, is a potent inhibitor of this protease, with a K_i_ value of 7.9 nM. Consequently, the present study describes, for the first time, the attainment and the biochemical characterization of a recombinant cystatin from leeches as a potent CatL inhibitor. While searching out for new molecules of therapeutic interest, this leech cystatin opens up possibilities for the future use of this molecule in studies involving cellular and in vivo models.

## 1. Introduction

Human cysteine proteases participate in several physiological processes, such as the degradation of peptides and proteins [1], and constitute the major components of lysosomes [2]. In this group of enzymes, cathepsin L (CatL) is an endopeptidase that degrades intracellular and endocytosed proteins in the lysosome. Recent studies have suggested that this protease plays many critical roles in diverse cellular settings. Thus, overexpression of CatL has been reported in several human diseases, such as liver fibrosis, Type I and II diabetes, cardiac, bone, immune, and kidney disorders [3,4,5]. Additionally, membrane-bound or released CatL mediates the cleavage of the S1 subunit of the corona-virus surface spike glycoprotein, participating in the invasion into human host cells via the so-called late pathway [6,7]. Although the preferred pathway for infection is via the serine protease TMPRSS2 (early pathway), CatL, together with TMPRSS2, present tempting targets for pharmacological inhibition [7]. 

The endogenous inhibitors named cystatins are the most effective mechanism for controlling the activity of cathepsin L [1,2]. It is interesting to note that CatL inhibitors can also be found in several organisms, such as hematophagous animals, where they play an important role in their survival. Cystatins from hematophagous animals participate in the immunological modulations [8], reducing the processing capacity and presentation of antigens by the host’s antigen-presenting cells. The production of cytokines by the host’s macrophages is also affected, resulting in an anti-inflammatory response [9]. Tick cystatins have been characterized as capable of inhibiting several cathepsins involved in blood digestion, embryonic development of the tick, and the immune response of the host [10,11]. Cystatins that specifically inhibit CatL have also been described, such as sialostatin L, present in the *Ixodes scapularis* tick. Sialostatin L inhibits the protective proteolytic activity of host cells at infestation sites, thus promoting tick survival [12]. In addition, it also holds anti-inflammatory and immunosuppressive activities through the inhibition of killer T cells [13].

Tick cystatins have been described as potent and specific protease inhibitors, expanding their potential to be used further as new vaccine antigens and anti-tick drugs of medical importance [10,14]. Furthermore, studies of cystatins in cell models, such as cancer [15], inflammation [16], and immunomodulation [17], demonstrate the significant potential of cystatins as new drugs or prototypes for developing new drugs for veterinary and human use.

Similar to ticks, leeches are hematophagous parasites that possess interesting compounds in their saliva capable of assisting the maintenance of blood flow for successful feeding. Thus, many anticoagulant and antiplatelet molecules have been characterized for this purpose [18,19,20,21]. However, cysteine protease inhibitors have rarely been studied in the literature, and, until now, no cystatin—native or recombinant—was characterized for leeches apart from the cystatin B gene first characterized in *Theromyzon tessulatum* leeches. It was demonstrated that the innate immune response in the leech involves a cysteine protease inhibitor not previously detected in other invertebrate models, highlighting the need for further study of the innate immunity mechanism in these animals [22].

The present work is the first of its kind to characterize the recombinant cystatin of leeches attained through the sequence of the Hviz00340 transcript from the transcriptome of *Haementeria vizottoi* [23]. Cystatin-Hv was successfully expressed in *Pichia pastoris*, and after purification, was characterized for its ability to inhibit cathepsin L. The present study of the first recombinant cystatin derived from leeches will allow a more detailed investigation of its role in feeding the parasite. In addition, the molecule itself can be further investigated in cellular and in vivo models to better understand its potential role in the search for new molecules of therapeutic interest.

## 2. Results

### 2.1. Selection and Purification of Cystatin-Hv

The cystatin-Hv cDNA sequence, coding for the predicted cysteine protease inhibitor derived from the leech *Haementeria vizottoi*, is composed of 396 nucleotides, resulting in 131 amino acids. The signal peptidase cleavage site of Cystatin-Hv predicted by SignalP and Expasy was located between the 19th and 20th amino acid residue. To express and secrete the recombinant protein in *Pichia pastoris*, the DNA fragment corresponding to the mature protein was cloned into pD912-AK. The calculated molecular mass of this predicted protein is 12,487.92 Da, and the pI is 5.23.

**Figure 1:**
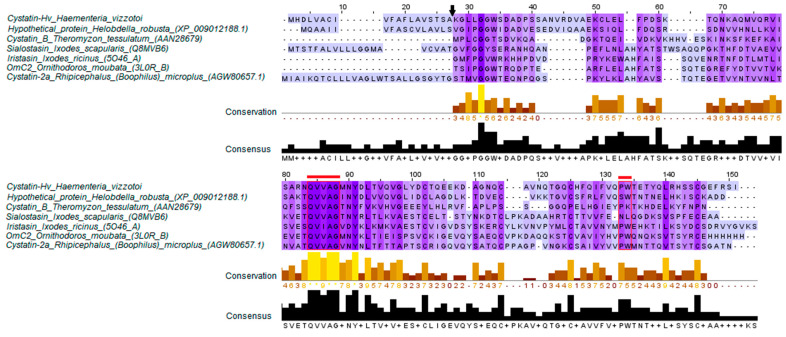
Multiple alignments of cystatin-Hv with other cystatins from leeches and ticks. Regions highlighted in red show the first hairpin loop and the second hairpin loop. The black arrow indicates the signal peptide cleavage site. The conservation of the sequences is shown in yellow bars, and the score values 9 and 10 (or asterisk) indicate total conservation of the aligned sequences; the consensus sequence is shown in black bars (Clustal Omega).

This protein presents domain and typical cystatin active sites, such as the highly conserved first hairpin loop QVVAG and the second hairpin loop PW [24], associated with cystatins type C (Figure 1). The highest identity is found with a hypothetical protein of the leech *Helobdella robusta* (47% identity, access number: XP_009012188.1), presenting low identity with the preliminary characterized cystatin B of the leech *Theromyzon tessulatum* (21% identity, access number: AAN28679) [25], the Sialostasin of the tick *Ixodes scapularis* (19% identity, access number: Q8MVB6) [26], the Iristasin of the tick *Ixodes ricinus* (14% identity, access number: 5O46_A) [16], the OmC2 of the tick *Ornithodoros moubata* (21% identity, access number: 3L0R_B) [27], and with the cystatin 2a of the tick *Rhipicephalus (Boophilus) microplus* (21% identity, access number: AGW80657.1) [28].

**Figure 2:**
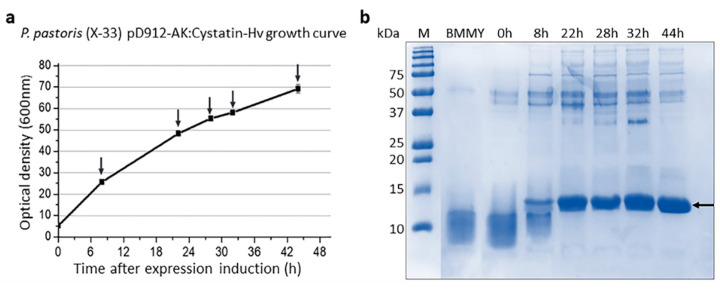
Growth curve of *P. pastoris* (X-33) pD912-AK: Cystatin-Hv during the assay of recombinant protein expression (**a**) and Coomassie-stained SDS-PAGE (15%) of proteins recovered from culture supernatant collected during the experiment (**b**). Vertical arrows: points of methanol feeding and supernatant collection; lane M: low molecular weight protein markers “Precision Plus Protein™ Dual Color Standards” (Bio-Rad); lane BMMY: fresh culture medium; lanes 0 h to 44 h: proteins recovered at different incubation times; horizontal arrow: expected sized protein band.

For the expression of the recombinant Cystatin-Hv, the selected expression vector carries a secretion signal derived from *S. cerevisiae* (SS alpha-factor), located upstream of the insert, which fused to the recombinant protein, promotes its secretion out of the cell. The production of Cystatin-Hv using *P. pastoris* (X33) was performed as described, with four 100 mL replicates, beginning the expression step with OD_600nm_ around 5, going up to OD_600nm_ 69 after 44 h of assay (average values), as shown in Figure 2.

**Figure 3:**
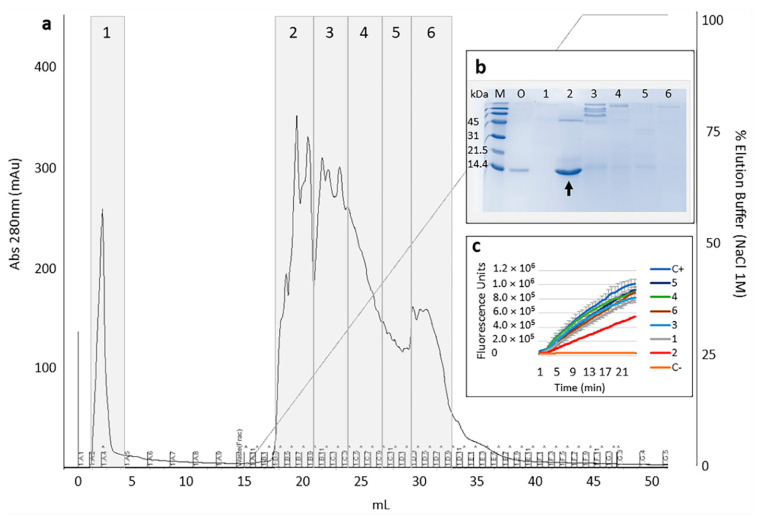
Chromatogram showing the elution profile of culture supernatant proteins from MonoQ resin, using 1 M NaCl as elution buffer, whose fractions were pooled 1–6 for analysis (**a**) SDS-PAGE of pooled fractions (**b**) and inhibition assay of papain activity (papain 10.7 nM, zFR-MCA substrate 5 µM) in the presence of protein pools (250 ng) (**c**). (**b**) Lane M: low molecular weight protein markers “SDS standards low range” (Bio-Rad); O: culture supernatant; lanes 1 to 6: proteins recovered from pools; vertical arrow indicates protein band compatible with Cystatin-Hv. (**c**) Here C+ indicates the positive control (papain and substrate), and C− indicates negative control (substrate).

**Figure 4:**
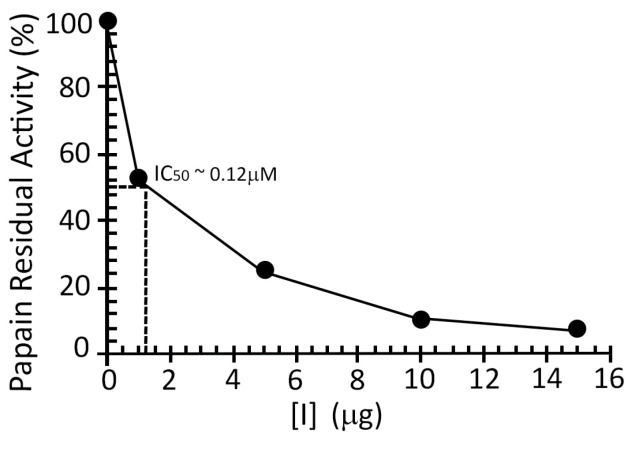
Inhibition assay of Cystatin-Hv (1 µg, 5 µg, 10 µg e 15 µg of pool 2 from ion-exchange chromatography) against papain (21.4 nmol/L or 50 ng) using 5 µM of Z-FR-MCA substrate. An estimate of the half maximal inhibitory concentration (IC_50_) is given, considering the dominance of Cystatin-Hv in pool 2.

Culture supernatant after 44 h of expression was recovered, concentrated, dialyzed, submitted to ion-exchange chromatography, and pooled fractions were analyzed on SDS-PAGE and used for inhibition assays against papain (Figure 3). Inhibitory activity was detected relative to pool 2 (eluted within 10% to 15% of NaCl 1 M buffer), evidenced by the decrease in fluorescence emission (the result of proteolysis) when compared to other pooled fractions and positive control (reaction without pooled fractions). Inhibition of papain occurred in a dose–response manner, as shown in Figure 4.

**Figure 5:**
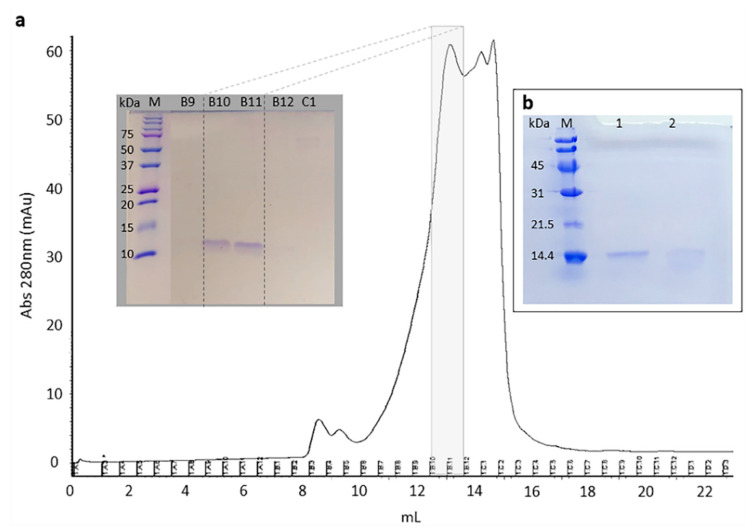
Size exclusion chromatography of pool 2 (from previous ion-exchange chromatography), highlighting protein bands into fractions B10 and B11 (purified Cystatin-Hv), on SDS-PAGE, related to inhibitory activity against papain and cathepsin L (**a**), SDS-PAGE analysis of purified Cystatin-Hv under reducing (1) and nonreducing (2) conditions (**b**). Molecular markers used were “Precision Plus Protein™ Dual Color Standards” (Bio-Rad) (**a**) and “SDS standards low range” (Bio-Rad) (**b**).

Once the inhibitory activity was detected, pooled fractions (referred to as pool 2) were further purified by size-exclusion chromatography, leading to a single band named Cystatin-Hv (Figure 5). Mass spectrometry analysis (LC-MS/MS) was performed to confirm the accuracy of the molecular mass and allowed the identification of Cystatin-Hv with eight unique peptides, covering 89% of the mature protein sequence (Supplementary Figure S1). 

### 2.2. Inhibition Studies

**Figure 6:**
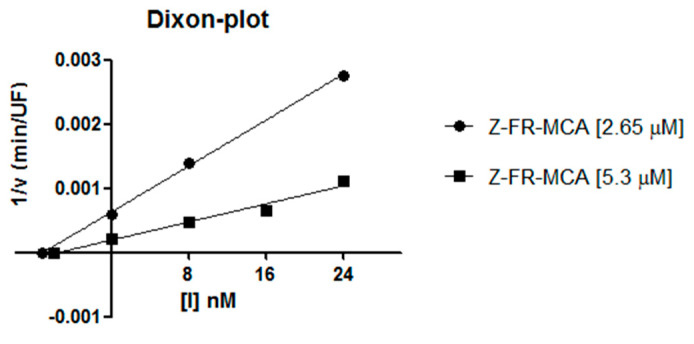
Dixon diagram presenting kinetic enzymatic assays of cathepsin L inhibition by Cystatin-Hv. Two substrate concentrations (1 K_m_ or 2.65 μM and 2 K_m_ or 5.3 μM) and three cystatin-Hv concentrations (8 nM, 16 nM, and 24 nM) were used. The inhibition constant was set as 7.9 nM. The graphic was made using the software GraphPad Prism 5. Slope value: 7.964 × 10^−5^ to 9.909 × 10^−5^ (2.65 μM) and 2.496 × 10^−5^ to 4.547 × 10^−5^ (5.3 μM) for a 95% confidence interval. UF: Units of Fluorescence.

Enzymatic kinetics assays were performed with three different concentrations of Cystatin-Hv (8 nM, 16 nM, and 24 nM respectively), cathepsin L (0.4 nM) and two concentrations of Z-FR-MCA substrate (1 K_m_ and 2 K_m_). Experimental data of reaction rates were linearized and treated further as proposed by Dixon [29] (Figure 6), thus allowing us to determine the inhibition constant, K_i_, of 7.9 nM.

## 3. Discussion

The present work was carried out to assess biodiversity and contribute to developing new molecules that can generate and inspire new therapeutic possibilities. In this context, the search for molecules from animal secretions related to feeding is rather compelling since, from the evolutionary perspective, the proteins present in such secretions have been subjected to selective pressure for better efficiency to ultimately facilitate the animal’s survival and perpetuation [30]. Hence, it is expected that proteins present in the saliva of hematophagous animals should have a specific action on the host or prey, and it is up to the researchers to isolate these components, identify their actions, and study how these molecules can be used for our benefit.

Cystatins present in hematophagous have the key function in inhibiting endogenous cysteine proteases of the animal and in helping the feeding process as well. The saliva of these animals contains not only inhibitors that reduce host blood clotting and premature blood clotting inside the gut but also molecules that interfere and inhibit the performance of the host’s immune system [17,24] and allow the hematophagous to keep feeding for an extended period. 

An important dimension in the discussion on cystatins present in the salivary complexes of leeches relates to the issue of the innate immune response. It is reported that symbiotic bacteria, antimicrobial peptides, and phagocytic immune cells play a protective role in defending from harmful agents and preventing premature degradation of the ingested blood meal, which is concentrated and maintained over a period of many weeks inside the digestive tract [31]. Although the leech defense system has been poorly investigated, studies with cystatin B have demonstrated the involvement of this cysteine protease inhibitor in the innate immunity of *Theromyzon tessulatum* leeches since an increase in cystatin B gene expression has been shown in large circulating coelomic cells after bacterial challenge [22,25]. While more studies are needed to further elucidate the function of cysteine protease inhibitors for leeches, it is likely that these molecules also work as immunoregulators, given the major implication of cathepsins in immunity [22], similarly to what has been described for ticks, a better characterized group of hematophagous.

In ticks, this group of inhibitors has been extensively explored. It was noted that in tick saliva, the majority (84%) of cystatin transcripts belong to a group that is secreted extracellularly, suggesting a predominantly immunoregulation function [32]. Cystatin OmC2, from the *Ornithodoros moubata* tick, for example, targets two lysosomal cathepsins, S and C, which perform the function of processing antigens in antigen-presenting cells, apart from affecting the maturation of dendritic cells [17]. Cystatin Iristatin, identified from the tick *Ixodes ricinus*, inhibited the proteolytic activity of cathepsins L and C and decreased the production of several inflammation inducers (IL-1, IL-4, IL-9, IFN-γ) by different populations of T cells, among other anti-inflammatory activities [16]. 

Cystatins are also present in humans, where, as in other animal species, they act as inhibitors of endogenous cysteine proteases, such as cathepsins. Overexpression of these enzymes has been observed in a number of tumorous cells, such as breast, lung, brain, head, neck, and melanoma cancers, where they act on the degradation of the extracellular matrix enabling tumor growth, invasion of other tissues, and migration into the bloodstream [2,8]. In particular, cathepsin L is a lysosomal endopeptidase widely expressed and involved in the degradation of intracellular or phagocyted proteins that can also be found in a variety of extracellular media as well as in the cell nucleus [33,34]. In this way, positive regulation of the lysosomal endopeptidase cathepsin L has often been observed in a number of human cancers, and its levels of expression in tumor tissues or their presence in the environment adjacent to the tumors is considered to be largely correlated with their aggressiveness [2,34,35,36].

There is little information available about cystatins regarding leeches, most of which are the results of transcriptomic analyses suggesting the participation of these molecules in the immune response [25]. Functional studies with cystatins present in the leeches have not yet been reported in the literature.

The present study started with the library of transcripts of the salivary complexes of the leech *Haementeria vizottoi*, where 1204 Isotigs were obtained, and among them, 123 were identified as related to feeding [23]. After further screening, one Isotig was selected for this study, starting with the gene sequence, through the cloning and recombinant production of the protein, Cystatin-Hv, to its functional characterization.

In general, the benefits of protein production by *P. pastoris* system include appropriate folding, especially for cysteine-rich proteins (in the endoplasmic reticulum) and secretion (by Kex2 as signal peptidase) of recombinant proteins to the supernatant environment of the expression [37]. In the case of Cystatin-Hv, a protein with five cysteines, the expression occurred satisfactorily, as expected, with compatible quality acceptable to the scalability of the process. Furthermore, the use of the *P. pastoris* expression system, due to its limited production of endogenous secretory proteins, is known to favor an easy purification protein process [37]. In this sense, the isolation in two chromatography steps was sufficient to achieve a pure form of recombinant Cystatin-Hv, similar to the purification process performed by Cardoso [38], characterizing a tick cystatin that presented an inhibitory effect against the activity of a hemoglobin lytic enzyme.

The inhibition assays allowed us to confirm the activity of Cystatin-Hv, in its recombinant form, as a strong inhibitor of cathepsin L. Further, results plotted in the Dixon diagram (Figure 6), with curves intercept on the X-axis, suggest a noncompetitive mechanism of action for this inhibitor. Although cystatins are usually described as competitive inhibitors, the noncompetitive mechanism was observed for soybean [39], corn [40], and chestnut seed [41] plant cystatins, as well as for human Cystatin SA [42]. In order to improve Cystatin-Hv characterization and understanding of its mechanism, complementary assays are to be performed, also against other known cathepsins. The inhibition constant (K_i_) in the order of nM (7.9 nM) is compatible with the one found in the literature for the dissociation constant of cathepsin L with human cystatins [1]. Similar K_i_ values were obtained related to cystatins of hematophagous animals such as the bovine ectoparasite *Rhipicephalus microplus*, whose protein identified as Rmcystatin-4 was cloned, expressed, and purified, and has demonstrated inhibitory activity against cathepsin L with a K_i_ of 11.1 nM [38]. The cystatin OmC2 from the tick genus *Ornithodoros* also presented similar K_i_ values in the range of nM against lysosomal cathepsins S and C [17].

Although the K_i_ value in relation to papain has not been obtained, the IC_50_ value of approximately 0.12 µM, considering Cystatin-Hv dominant in pool 2, indicates a greater potency of cystatin-Hv for the inhibition of cathepsin L. However, future studies should be carried out with papain and other cathepsins to assess the specificity of cystatin-Hv in relation to a particular protease.

The character of recombinant Cystatin-Hv as an inhibitor of cysteine proteases, especially human cathepsin L, opens interesting possibilities for its potential biological function as an immunoregulator and an anti-inflammatory molecule, justifying our efforts to study this protein in its recombinant form. Inhibition of CatL has also been recognized as having a significant role in the prevention of cell invasion by viruses of the coronavirus family in vitro. Given the recent emergence of the novel SARS-CoV-2, calls for more attention to inhibitors of this cysteine protease are well justified [7]. Thus, the first recombinant cystatin from leeches will allow a more detailed investigation of its role in feeding the parasite. In addition, the molecule itself can be investigated in cellular and in vivo models to understand its significance in the possible search for new molecules of therapeutic interest.

## 4. Conclusions

The present work is the first of its kind to characterize the recombinant cystatin of leeches attained through the sequence of the transcript Hviz00340 from the transcriptome of *Haementeria vizottoi* [23]. Cystatin-Hv was successfully expressed in *Pichia pastoris,* and after purification, it was characterized for its ability to inhibit cathepsin L. Kinetic studies have indicated that recombinant Cystatin-Hv is a potent inhibitor of cathepsin L, with a K_i_ of 7.9 nM. Thus, a rigorous study of this molecule could be promising, and future work will be carried out in this direction to better characterize the therapeutic potential of Cystatin-Hv.

## 5. Materials and Methods

### 5.1. Strains, Plasmids, Enzymes

The expression vector pD912-AK with the synthesized insert of interest (codon-optimized) was purchased from ATUM 2.0 (Newark, NJ, USA), and *P. pastoris* strain X-33 (Invitrogen, Waltham, MA, USA) was used as the expression host. The *Escherichia coli* strain DH5α and restriction enzyme SacI were purchased from Thermo Fisher (Waltham, MA, USA). *E. coli* cells with plasmids were cultured at 37 °C in Luria–Bertani medium (yeast extract, 5 g/L; tryptone, 10 g/L; NaCl, 10 g/L; agar, 15 g/L) containing 25 μg/mL Zeocin (Invitrogen, Waltham, MA, USA). Papain was purchased from Sigma-Aldrich (St. Louis, MO, USA) and human cathepsin L from R&D Systems (Minneapolis, MN, USA).

### 5.2. Sequence Source and In Silico Characterization

The Cystatin-Hv cDNA sequence (transcript Hviz00340) was obtained from the sialotranscriptome of *Haementeria vizzotoi* leech [23]. The signal peptide sequence, determined by SignalP 4.0 [43], was excluded from further analysis and from the insert synthesis. Theoretical pI and Mw were determined using the Expasy platform. Identity and similarity percentages of the full-length amino acid sequence were obtained by BLAST search (NCBI database), and multiple sequence alignments were performed on a sequence of Cystatin-Hv versus known cystatins, using Clustal Omega [44].

### 5.3. Expression and Purification of Recombinant Protein

The vector pD912-AK: Cystatin-Hv was linearized with SacI and electroporated into competent *P. pastoris* X-33 cells. Transformants were screened on YPD medium plates containing 25 μg/mL Zeocin, and the presence of Cystatin-Hv insert was confirmed by PCR. Expression was carried out in replicates inoculating 50 mL of BMGY medium [1.0% yeast extract, 2.0% peptone, 100 mM potassium phosphate pH 6.0, 1.34% YNB, 4 × 10^−5^% D-biotin (*w*/*v*), and 1% glycerol (*v*/*v*)] and cultivated under the influence of 350 rpm orbital shaking at 28 °C for 24 h. Cells were harvested by centrifugation at 450× *g* for 5 min at 4 °C and resuspended in 50 mL of BMMY medium [1.0% yeast extract, 2.0% peptone, 100 mM potassium phosphate pH 6.0, 1.34% YNB, 4 × 10^−5^% D-biotin (*w*/*v*), and 0.5% methanol (*v*/*v*)] to absorbance at 600 nm of 5.0. Incubation was carried out at 30 °C and 350 rpm orbital shaking for 44 h, with further additions of methanol to a final concentration of 0.5% every 12 h, approximately. Samples (1 mL) were taken during the assay, submitted to protein precipitation with methanol/chloroform [45], and analyzed by SDS-PAGE. 

Cells were removed from the supernatant by centrifugation (3500× *g* for 15 min at 4 °C) and filtration (0.45 μm). The supernatant was dialyzed (5 kDa molecular exclusion) and concentrated with 20 mM Tris-HCl pH 8.0 using Cogent µScale TFF System (Merck, Darmstadt, Germany) and submitted to ion-exchange chromatography in a Mono Q 5/50 GL (GE Healthcare) 1 mL column connected to an AKTA Avant system (GE Healthcare), equilibrated with 20 mM Tris-HCl pH 8.0. The sample was added, and the column was washed with 15 CV (column volume) of 20 mM Tris-HCl pH 8.0 (0.5 mL/min), followed by the elution step supported by a crescent linear gradient of 20 mM Tris-HCl pH 8.0, 1.0 M NaCl along 30 CV (0.5 mL/min). Fractions (~500 μL) were pooled, analyzed by SDS-PAGE, and the one presenting inhibitory activity towards papain was applied on Superdex 75 10/300 column (GE Healthcare), being eluted with 20 mM Tris-HCl pH 8.0 along 2 CV (1 mL/min). Fractions with expected molecular weight, single band, were pooled, quantified using the bicinchoninic acid (BCA) Protein Assay Kit (Pierce, WA, USA) and further analyzed for inhibitory activity against papain and cathepsin L.

### 5.4. Mass Spectrometry for Sequence Confirmation

A purified sample of the recombinant Cystatin-Hv was submitted to in-solution trypsin digestion prior to mass spectrometry analysis by LC-MS/MS. The generated tryptic peptides were desalted, dried, and dissolved in 20 µL of 0.1% (*v*/*v*) formic acid, and 2 µL were automatically injected into a 2 cm C-18 trap column (3 μm particle size, 100 Å pore size, 75 μm I.D., Thermo Fisher Scientific, Waltham, MA, USA) by an Easy nanoLC 1200 coupled to a QExactive plus (Thermo Fisher Scientific, Waltham, MA, USA) mass spectrometer. Chromatographic separation of tryptic peptides was performed on a 15 cm long analytical column (Acclaim PepMap, 2 µm particle size, 100 Å pore size, 50 µm I.D.—Thermo Fisher Scientific, Waltham, MA, USA). Peptides were eluted with a linear gradient of 5–100% Buffer B (80% acetonitrile in 0.1% formic acid) at 200 nL/min for 30 min. The spray voltage was set to 2.4 kV, and the mass spectrometer was operated in positive, data-dependent mode, in which one full MS scan was acquired in the m/z range of 300–1500 followed by MS/MS acquisition using high-energy collisional dissociation (HCD) of the seven most intense ions from the MS scan using an isolation window of 2.0 *m*/*z*.

The obtained MS and MS/MS spectra were analyzed using PEAKS Studio X, and the searches were performed against a customized database. Briefly, the database used included all *Pichia pastoris* protein sequences downloaded from UniProt (a total of 16,348 sequences, downloaded on 14 October 2021) and the translated amino acid sequence of Cystatin-Hv (without the signal peptide sequence). This reference database was concatenated with common contaminants for mass spectrometry experiments (116 sequences), and the decoy sequences were used for false discovery (FDR) rate control. The search engine was set to detect specific tryptic peptides at an FDR of 1%, allowing two missed cleavages. Methionine oxidation, acetylation of the protein N-termini, and deamidation of asparagine and guanidine were set as variable modifications, and carbamidomethylation of cysteine was set as a fixed modification.

### 5.5. Inhibitory Assays

Papain was utilized to select the chromatographic fractions that contained Cystatin-Hv, and, after obtaining the inhibitor in its homogeneous form, cathepsin L was used to determine the value of the inhibition constant (K_i_). The assays were implemented according to Portaro et al. (2000) [46] with some minor modifications. Enzymes were preactivated for 15 min at room temperature with 6 mM DTT in 50 mM sodium phosphate, 200 mM NaCl, 5 mM EDTA, and pH 5.5 (final volume 100 μL). For the pool selection steps, 10 ng of papain, 5 µM of fluorogenic substrate Z-FR-AMC (Sigma-Aldrich, St. Louis, MO, USA) and 250 ng of protein from pooled purification fractions were used. Cathepsin L (0.4 nM) was employed against three concentrations of purified Cystatin-Hv (8 nM, 16 nM and 24 nM) and two concentrations of the fluorogenic substrate Z-FR-AMC (1 K_m_ and 2 K_m_, where the K_m_ = 2.6 µM, ref. [47]) to determine the K_i_ value [48]. Control reactions were carried out in the same conditions but without Cystatin-Hv. The activity was measured (fluorescence at λ_EM_ 480 nm and λ_EX_ 360 nm) in a Victor 3 (Perkin Elmer, Boston, MA, USA) plate reader. The temperature remained constant at 37 °C, and one reading per minute was performed for 15 min, the plates being shaken before each measurement. The residual activity of human cathepsin L in the presence of Cystatin-Hv in different amounts was determined, and the inhibition constant (K_i_) of Cystatin-Hv towards human cathepsin L was determined by the Dixon Plot equation (1/V vs. [I]) [49], using the software GraphPad Prism 5.

## Supplementary Materials

The following are available online at https://www.mdpi.com/article/10.3390/toxins13120857/s1, Figure S1: Amino acid sequence of the mature Hviz340 recombinant protein and the tryptic peptides identified by LC-MS/MS analysis using a QExactive plus mass spectrometer and database search in PEAKS Studio X.
