# Supplementary material for: Novel Cysteine Protease Inhibitor Derived from the Haementeria vizottoi Leech: Recombinant Expression, Purification, and Characterization
Source: Toxins (Basel). 2021 Dec 2;13(12):857. doi: 10.3390/toxins13120857 (PMC8705320; doi:10.3390/toxins13120857)
Supplement: Supplementary file 1 [file toxins-13-00857-s001.zip › toxins-1426630-supplementary.pdf]

toxins

Supplementary material: Novel cysteine protease inhibitor derived from the *Haementeria vizottoi* leech: recombinant expression, purification and characterization

Débora do Carmo Linhares, Fernanda Faria, Roberto Tadashi Kodama, Adriane Michele Xavier Prado Amorim, Fernanda Calheta Vieira Portaro, Dilza Trevisan-Silva, Karla Fernanda Ferraz and Ana Marisa Chudzinski-Tavassi

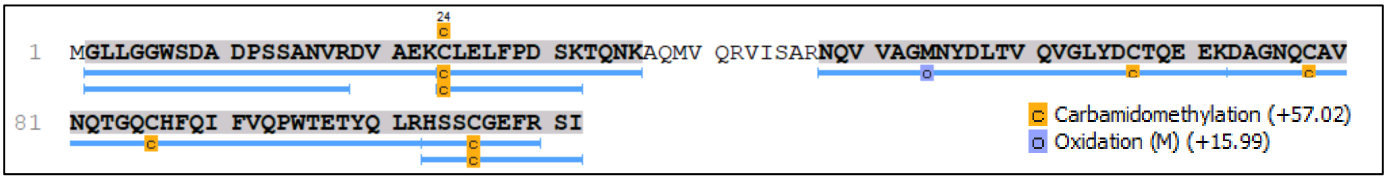

Figure S1: Amino acid sequence of the mature Hviz340 recombinant protein and the tryptic peptides identified by LC-MS/MS analysis using a QExactive plus mass spectrometer and database search in PEAKS Studio X. Blue lines correspond to the identified tryptic peptides. The protein was identified with 8 unique peptides, covering 89% of the mature protein sequence.
